# International journal of mental health systems: a bibliometric study

**DOI:** 10.1186/1752-4458-8-1

**Published:** 2014-01-06

**Authors:** Harry Minas, Alexandra Wright, Mengxue Zhao, Ritsuko Kakuma

**Affiliations:** 1 Centre for International Mental Health, School of Population and Global Health, The University of Melbourne, 3010 Parkville, Victoria, Australia; 2 International Journal of Mental Health Systems, Parkville, Australia

## Abstract

**Background:**

The International Journal of Mental Health Systems (IJMHS) was launched in August 2007 and has recently been given a formal impact factor. This study uses bibliometric indicators to review the performance of the Journal against its original stated objectives and aspirations.

**Methods:**

All articles published in IJMHS since publication commenced were included (n = 158). Selected bibliometric measures indicating Journal productivity, author affiliation, impact, geographic reach, and international collaboration were utilised.

**Results:**

IJMHS published 158 articles in seven volumes over six years. Articles with three to five authors constitute the dominant authorship pattern, and authors’ affiliations are varied. IJMHS has received an impact factor of 1.06 from Thomson Reuters, and the SCImago Journal Ranking shows IJMHS to be well positioned in the four categories in which it is listed, including in comparisons with well-established BMC journals that have similar scientific interests. Geographic authorship patterns show contributions from a large number of countries, including many low- and middle-income countries.

**Discussion:**

Manuscript submissions from a wide range of countries, including low- and middle-income countries, are mostly from academic institutions. Authors from some geographic areas of the world are significantly under-represented. The calculation of an impact factor and encouraging rankings on the SCImago Journal Rank index are expected to lead to increased submission of high quality manuscripts.

**Conclusion:**

The performance of IJMHS over the first six years is promising, and the Journal is on the way to achieving the aims set out in the inaugural Editorial. IJMHS will continue to enhance its current impact through a number of new initiatives, including the introduction of thematic series and a broader range of article categories.

## Background

The International Journal of Mental Health Systems was launched in August 2007. In the Editorial announcing the launch of the journal, the purpose of IJMHS was outlined as follows: “The *International Journal of Mental Health Systems* aims to stimulate greater attention to the central importance of building functioning mental health systems… We intend that this will be the journal to which mental health system researchers, Health Ministers’ advisers, policy makers, mental health consultants advising countries on mental health system development, teachers in psychiatry, nursing, psychology, social work and public health courses, clinicians involved in mental health system reform, and others will turn for the latest research and policy information on how to build equitable, accessible, efficient, high quality mental health systems” [1].

IJMHS is published exclusively online by BioMed Central as one of an increasing number of BMC independent journals. IJMHS is an open-access journal, and all publications are freely available at no cost.

In July 2013, on the sixth anniversary of commencement of publication, IJMHS received a formal impact factor for the first time. This study provides a review of the Journal’s performance against the aspirations outlined in the first editorial.

## Methods

The objective of this study is to review the performance of IJMHS against the Journal’s original aims as outlined in the inaugural editorial.

This review utilises bibliometric methods [2,3] to provide an overview of the journal’s influence, maturity, productivity, and network [4]. Numerous single-journal bibliometric studies have been performed previously, and reviews of these studies provide extensive lists of bibliometric measures available for use [2,5,6]. The bibliometric indicators selected for this article are intended to give a broad overview of the performance and impact of IJMHS from its inception up to July 2013.

The study was based on publically available information and did not require ethics approval.

### Sampling

All articles published by IJMHS (n = 158) were collected from the BioMed Central (BMC) archives from establishment in August 2007 to July 17, 2013.

### Data collection

The following descriptive data were collected for each article: year of publication; all authors’ names; authors’ institutional affiliations; and authors’ countries.

The impact of IJMHS was assessed using three indicators:

1. Electronic reference databases in which the journal is indexed.

2. Thomson Reuters Impact Factor: Measures of journal ‘prestige’ or influence are in the form of Impact Factors and rankings. The Thomson Reuters impact factor “is a measure of the frequency with which the ‘average article’ in a journal has been cited in a particular year or period”, and is the result of a ratio between the number of citations and recent citable items that have been published [7]. The impact factor for IJMHS has been supplied to the Editorial board, is advertised on the IJMHS website at BMC, and is reported here.

3. Journal Rank: As an alternative to the Impact Factor, the SCImago Journal and Country Rank is an online, open-access tool that provides journal rankings based on information drawn from the Scopus (Elsevier) research database. The SCImago Journal Rank (SJR) indicator builds upon the PageRank algorithm developed by Google, and considers both the number of citations a journal receives and the ‘prestige’ of the journals where these citations are located. In other words, the SJR indicates the importance of the journal and its articles as nodes in the broader literature network. This measure is not influenced by the size of the journal, and was developed to enable comparative analyses between journals. SCImago lists IJMHS in four categories. The SJR of IJMHS for each category, as well those for three comparison journals from BMC (BMC Psychiatry, BMC Public Health, BMC Health Services Research) that are listed in the same SCImago categories as IJMHS, have been collected from the *SCImago* website [8].

### Data management and analysis

All citations and full articles were collected in the citation manager Endnote X6. Each citation was subsequently exported into Microsoft Excel, where counts and calculations were performed. Descriptive analyses were conducted to measure IJMHS’ performance:

a) Number of papers published each year.

b) Number of authors per paper.

c) Authors’ institutional affiliations.

d) Thomson Reuters Impact Factor.

e) SCImago Journal Rank (SJR) in 4 categories; compared with 3 well-established BMC journals.

f) Geographic distribution of all authors by country.

g) Geographic distribution of first authors by country.

h) Analysis of international collaboration.

The number of articles published each year was recorded. To determine the average number of authors per article, an average was calculated for all articles published in IJMHS. In determining the total number of authors who have published in IJMHS, each individual author was only counted once regardless of the number of papers they published in IJMHS.

To analyse the geographic distribution of authors the country location of each author’s primary institutional affiliation was recorded.

To understand the institutional affiliation profile of IJMHS authors, the type of affiliation for each author was recorded. After a preliminary review of author affiliations, seven categories were developed and agreed upon by the research team: academic institution; hospital/public mental health service; research institute; government department or agency; international organisation; non-governmental organisation (NGO) or association; and private company. For each author, all stated affiliations were recorded and categorised, i.e. if they had more than one stated affiliation they were categorised in all relevant categories. The percentage of authors in each category was calculated and is depicted below.

To analyse the level of international collaboration between IJMHS authors, the percentage of articles published with authors from two or more countries was calculated for each year.

## Results

From establishment in August 2007 to 17 July 2013 IJMHS had published 158 articles in seven volumes.

The full text of all research articles is deposited in PubMed Central, the US National Library of Medicine’s full-text repository of life science literature, and other digital archives including e-Depot (The Netherlands). Other bibliographic databases that index articles published in IJMHS include: Citebase, DOAJ, Embase, Google Scholar, Index Copernicus, OAIster, PsycINFO, PubMed, PubMed Central, SCImago, Scirus, Social Sciences Citation Index, SOCOLAR and Zetoc.

**Figure 1:**
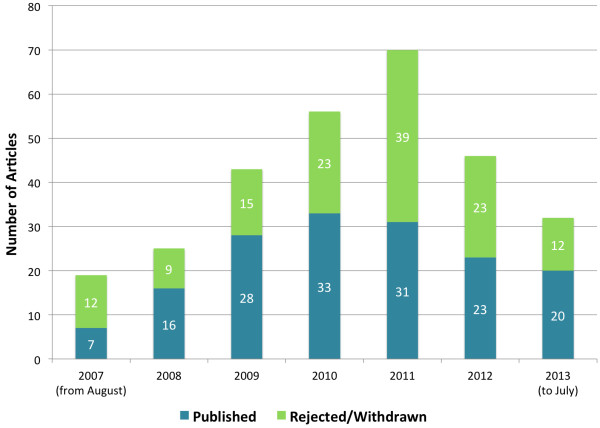
IJMHS manuscript submissions, publications and rejections by year.

Manuscript submissions to IJMHS increased each year from 2007 to 2011, with a decline in 2012. (Figure 1) Although there was a decrease in number of manuscripts published in 2011 and 2012 the submission rate picked up again in 2013.

**Figure 2:**
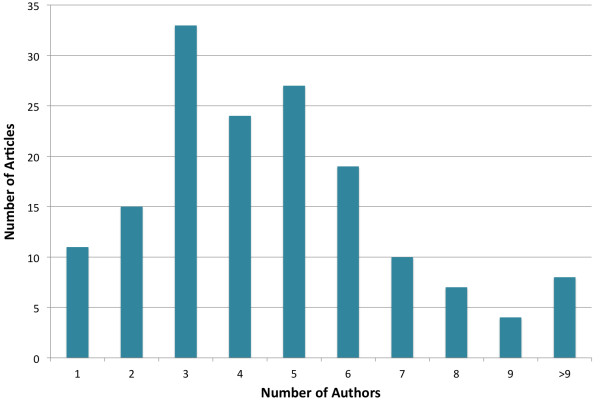
Number of articles by number of authors in each year.

Articles had an average of 4.7 authors. Figure 2 shows the number of articles by number of authors per paper. The most common number of authors was three (n = 33 articles), with the majority of articles ranging from three to six authors per article.

**Figure 3:**
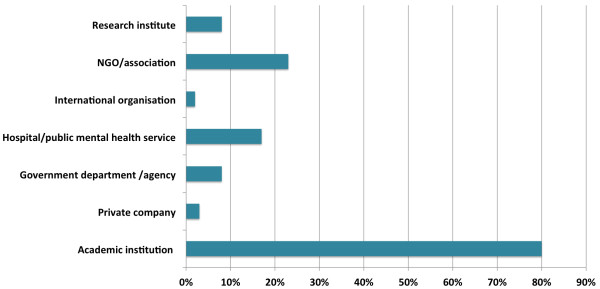
**Percentage of authors in each category of affiliation.** Legend: The numerator is number of author affiliations in a given category (i.e. authors with affiliations in different categories were counted in more than one category), and the denominator is the total number of IJMHS authors (i.e. authors were only counted once). The total percentages therefore sum to greater than 100%.

Authors of IJMHS articles are affiliated with a wide range of institutional types (Figure 3). As expected, the proportion of authors affiliated with academic institutions, such as universities or colleges, accounted for the largest category of institutions with which authors are affiliated, followed by non-government organisations/associations and hospitals/public mental health services, research institutes, government departments/agencies, companies, and international organisations.

**Figure 4:**
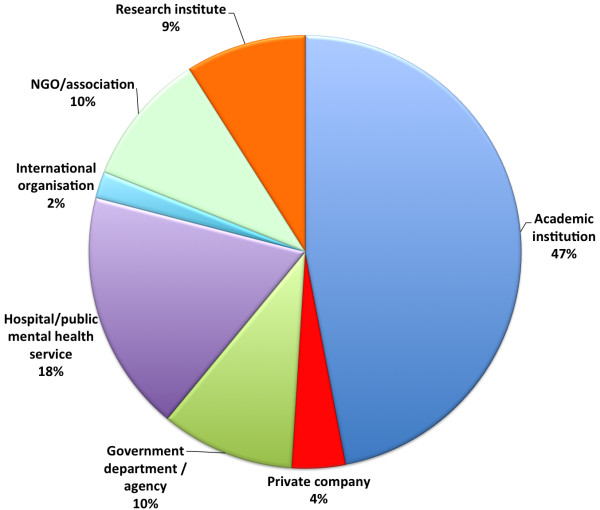
Percentage of each institutional category of affiliation (n = 270).

As shown in Figure 4, the category of academic institutions constituted the largest number of unique institutions with which authors were affiliated (n = 128), followed by hospitals and public mental health services (49), non-governmental organisations or associations with (28), government departments or agencies (26), research institutes (23), private companies (11) and international organisations (5).

Thomson Reuters now tracks IJMHS, with the first formal impact factor (1.06) announced in July 2013 [7]. As an alternative to the impact factor, the SCImago Journal Rank (SJR) lists IJMHS in four categories: Health Policy; Psychiatric Mental Health; Psychiatry and Mental Health; and Public Health, Environmental and Occupational Mental Health [8]. The SCImago rankings for IJMHS in the four categories, and the top-ranking journal in each category, are shown in Table 1.

**Table 1:** IJMHS rank in the four SCImago categories in which it is listed

***SCImago category***	***Highest ranked***	***Number of journals***	***IJMHS***	***IJMHS***
	***journal in category***	***in category***	***rank***	***percentile***
Health Policy	Health Affairs	123	35	72.4
Psychiatric Mental Health	World Psychiatry	18	2	94.4
Psychiatry and Mental Health	Archives of General Psychiatry	329	97	70.8
Public Health, Environmental and Occupational Mental Health	Annual Review of Public Health	337	100	70.6

The SJR enables comparative analyses of journals. In Table 2 IJMHS is compared with three BMC journals with which it has overlapping scientific interests and which are listed in the same SCImago categories as IJMHS (BMC Psychiatry, BMC Public Health and BMC Health Service Research) using the SJR metric (2011) and impact factor (2012).

**Table 2:** Comparison of IJMHS with three BMC journals

**Journal**	**Year of**	**Total number**	**SJR (2011)**	**Impact**
	**journal**	**of publications**		**factor**
	**launch**	**by July 17, 2013**		**(2012)**
BMC Psychiatry	2001	1187	1.093	2.23
BMC Public Health	2001	5443	0.886	2.08
BMC Health Services Research	2001	2549	0.886	1.77
IJMHS	2007	158	0.618	1.06

These three journals are flagship Biomed Central publications, while IJMHS is one of an increasing number of independent journals published by BioMed Central. To provide further context for these journal rankings, in the SCImago category ‘Health Policy’ , BMC Health Services Research is 18 of 123 journals. The highest-ranking journal in the category is Health Affairs, with an SJR of 3.082. In the SCImago Psychiatry and Mental Health category BMC Psychiatry is ranked 51 of 329 Journals. The highest-ranking journal in this category is Archives of General Psychiatry, with an SJR of 5.559. BMC Public Health is ranked 51 of 337 journals in the SCImago category Public Health. The highest-ranking journal in the category is the Annual Review of Public Health, with an SJR of 4.261.

**Figure 5:**
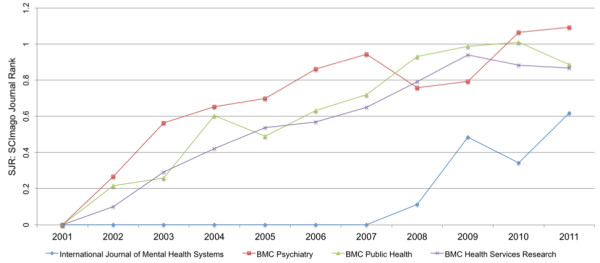
SJR in each year of publication of four BMC journals.

Figure 5 shows the SJR for each journal in each year since commencement of publication. All three of the BMC journals commenced publication in 2001, while IJMHS commenced publication in 2007.

**Figure 6:**
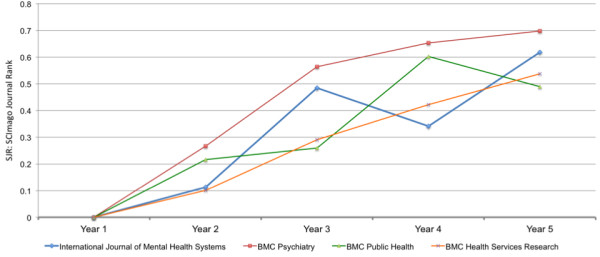
SJR for four BMC journals for the first five years of publication of each journal.

Figure 6 presents the SJR for the first five years of publication of each journal to get an indication of how IJMHS is tracking compared to the other journals during the initial years of publication. The pattern of increase in SJR in the first five years is broadly similar for IJMHS and the other three BMC journals.

**Figure 7:**
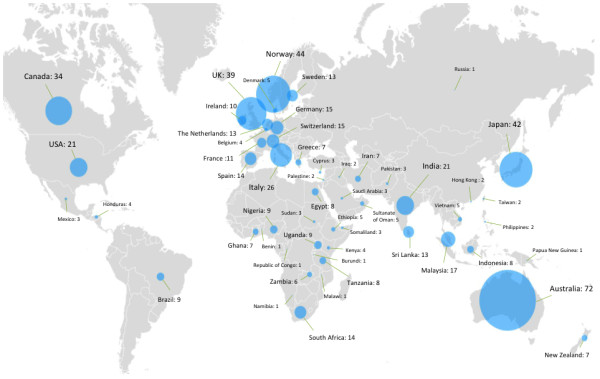
Country of authors for all IJMHS papers published since establishment of the journal.

**Figure 8:**
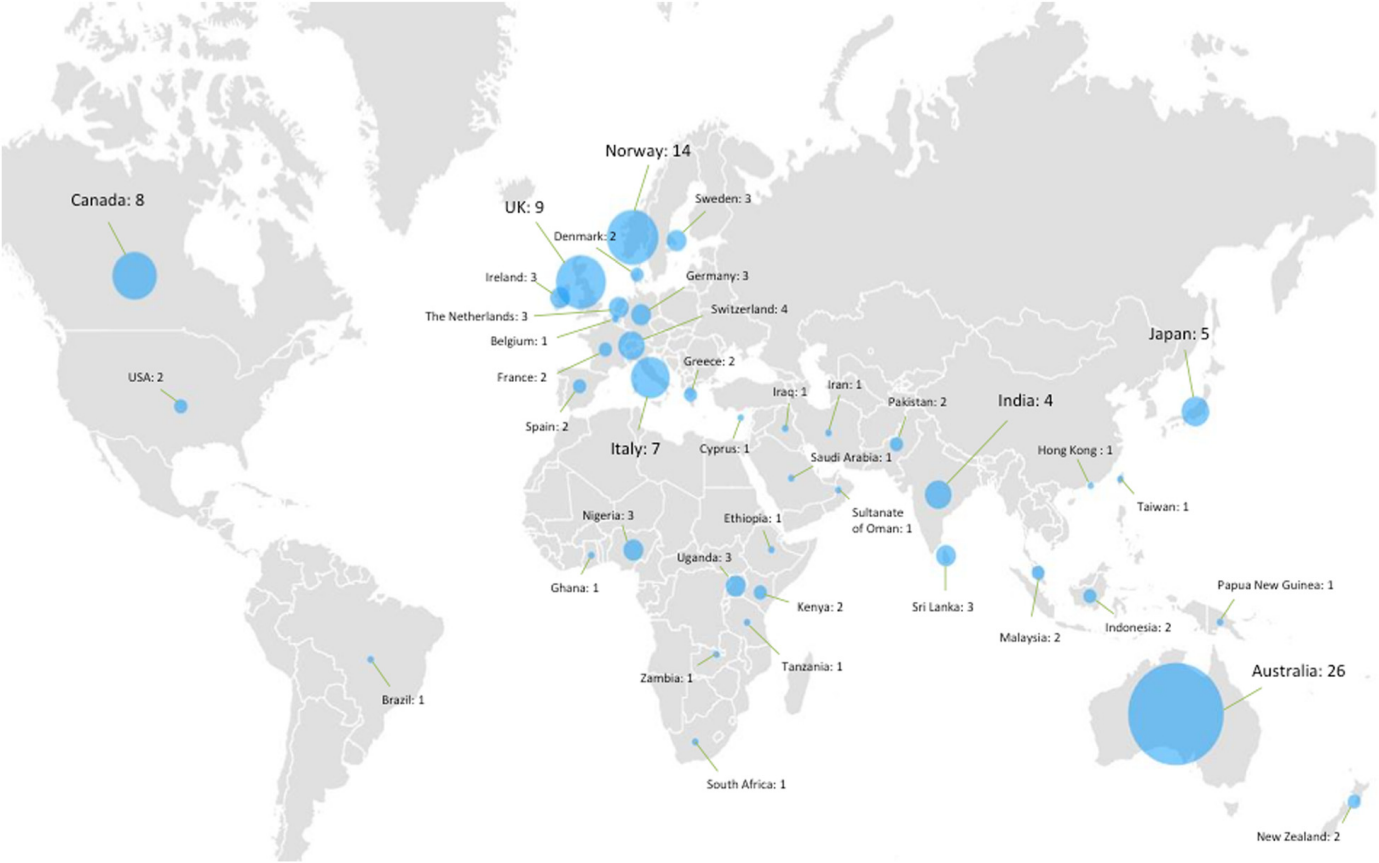
Country of first authors for all IJMHS papers published since establishment of the journal.

IJMHS aims to publish high quality papers from all parts of the world, particularly from low and middle-income countries, which are under-represented in the scientific literature. We have reviewed the countries from which all IJMHS authors come and, more particularly, the geographic spread of first authors, shown in Figures 7 and 8, respectively.

Geographic authorship patterns show a strong presence from Australia, Western Europe, North America, and Japan. Contributions have also been made from a number of African countries. South America, Eastern Europe, the Middle East and China are substantially under-represented.

First-authorship analysis shows a different geographic pattern than the total authorship analysis. Although the Australian and Western European presence remains strong, the presence of authors from North America, Africa, and Japan diminishes significantly when focusing on first-authorship.

**Figure 9:**
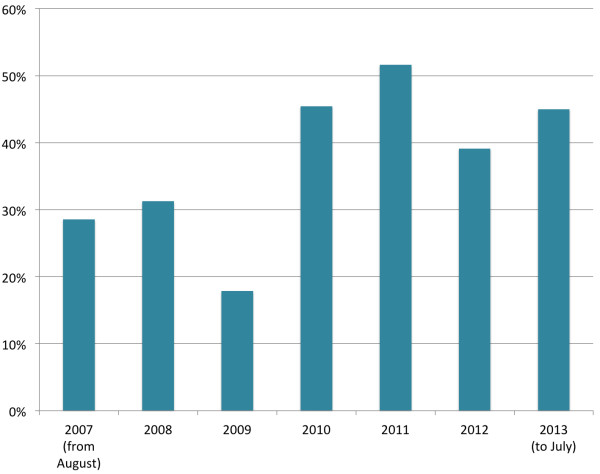
Percentage of papers with authors from more than one country by year.

In terms of international collaborations, 40% of IJMHS articles (n = 63) had authors from more than one country, with an increase in the latter four as compared to the first three years of publication (Figure 9).

Authors from Australia most frequently collaborate with authors from East and South Asia (Indonesia, Vietnam, Taiwan, the Philippines and India). Authors from Australia and the UK are more often represented in papers with multi-country authorship than are authors from other countries. Of papers that have authors from two or more countries many show collaboration between authors in high-income and low- or middle-income countries as defined by the World Bank Country Categories [9]. In total, 40 papers (25.3%) show author collaboration across country income categories. 18 papers (11.4%) show international collaboration only between high-income country authors and 10 papers (6.3%) include authors from more than one low- or middle-income country.

Table 3 shows the 20 most highly accessed papers since publication began. Half of the 20 papers have a first author from a low- or middle-income country. These 20 papers in total have been accessed by more than a quarter of a million readers, with a mean number of accesses of 13,234. The mean number of Pubmed citations is 5.6, and the mean number of Google Scholar citations is 29.8.

**Table 3:** 20 most highly accessed papers

**Paper**	**Country of**	**Year of**	**Accesses**	**PubMed**	**Google**
	**first author**	**publication**		**citations**	**scholar**
					**citations**
Reducing stigma and discrimination: Candidate interventions [10].	United Kingdom	2008	27,954	14	83
Cotard’s syndrome and delayed diagnosis in Kashmir, India [11].	India	2008	22,972	8	0
Collective trauma in northern Sri Lanka: a qualitative psychosocial-ecological study [12].	Australia/Sri Lanka	2007	19,834	8	58
Hope, despair and transformation: Climate change and the promotion of mental health and wellbeing [13].	Australia	2008	19,780	3	118
Relation between depression and sociodemographic factors [14].	Canada	2007	15,166	11	70
An overview of Uganda’s mental health care system: results from an assessment using the world health organization’s assessment instrument for mental health systems (WHO-AIMS) [15].	Uganda	2010	13,437	5	13
The mental health system in Brazil: Policies and future challenges [16].	Brazil	2008	12,502	0	31
Collective trauma in the Vanni- a qualitative inquiry into the mental health of the internally displaced due to the civil war in Sri Lanka [17].	Sri Lanka	2010	12,271	3	15
Job stress among community health workers: a multi-method study from Pakistan [18].	Pakistan	2008	11,837	6	31
Community mental health in India: A rethink [19].	India	2008	11,571	4	14
Three models of community mental health services In low-income countries [20].	United Kingdom	2011	10,874	3	14
*Pasung*: Physical restraint and confinement of the mentally ill in the community [21].	Australia	2008	10,372	10	23
Mental health policy in Kenya -an integrated approach to scaling up equitable care for poor populations [22].	Kenya	2010	10,304	11	26
Evaluation of psychological support for victims of sexual violence in a conflict setting: results from Brazzaville, Congo [23].	France	2009	10,279	6	21
Medication management and practices in prison for people with mental health problems: a qualitative study [24].	United Kingdom	2009	10005	1	13
Trauma-related psychological disorders among Palestinian children and adults in Gaza and West Bank, 2005–2008 [25].	France	2009	9,642	7	30
Parental HIV/AIDS status and death, and children’s psychological wellbeing [26].	United Kingdom/Norway/Ghana	2009	9,146	6	15
The Australian mental health system: An economic overview and some research issues [27].	Australia	2008	9,129	1	5
Mental health treatment outcomes in a humanitarian emergency: a pilot model for the integration of mental health into primary care in Habilla, Darfur [28].	Switzerland	2009	8,805	1	3
Human rights of persons with mental illness in Indonesia: more than legislation is needed [29].	Indonesia	2009	8,805	4	12
Totals and (means)			264,685	112 (5.6)	595 (29.8)

## Discussion

The editors of IJMHS are committed to online, open-access, peer-reviewed publication of research. The primary reason for this commitment [1] is to make the results of research freely available to the users of research - clinicians, service managers, policy makers, consumer and carer advocates, mental health NGOs and researchers – particularly in low and middle-income countries where there are financial and other barriers to access to research journals requiring subscription. In addition, researchers submitting their manuscripts to open access journals, and their institutions, benefit from the increased visibility, use and impact that open-access provides [30].

There is a general but modest upward trend in the number of manuscript submissions over the six years (Figure 1), with the exception of 2012, suggesting growing international acceptance of IJMHS as a journal of choice for research on mental health systems and development.

The wide range of authorship affiliations (Figures 3 and 4) is welcome. Although it is not surprising that more than half of all authors are affiliated with an academic institution or a research institute there is a substantial and increasing representation of authors affiliated with NGOs, mental health services and government.

A good geographic spread exists of all IJMHS authors (Figures 8 and 9). Representation of contributors from Africa and South-East Asia to IJMHS articles is comparable to that found in the wider mental health literature [31]. While the geographic spread of first authors of IJMHS articles is pleasing, there is room for considerable improvement in representation of a number of world regions and specific countries, particularly from the USA and South America, Eastern Europe and China. We particularly hope to see an increase in first authors from low and middle-income countries where considerable work is being done to strengthen mental health systems research capacity.

Having an impact factor from Thomson Reuters will have a positive influence on the journal since this is a common criterion for selection of journal by many authors and institutions [32]. The comparison of IJMHS on SJR with three well-established BMC journals during their first five years of publication (Figure 6) suggests that IJMHS is tracking well in building its level of impact.

The International Journal of Mental Health Systems is achieving the aims set out in the inaugural Editorial. IJMHS is receiving increased submissions from across the globe and from authors with a wide range of institutional affiliations, and is gaining international acceptance as a journal in which to publish high quality mental health system development papers. The receipt of an impact factor and respectable SJR results support this. The aim to be the journal where mental health policymakers, researchers, users and other stakeholders find information they can use to improve the performance of mental health systems [1] continues to be the aspiration.

## Conclusion

These are encouraging initial results. IJMHS intends to build upon the observed momentum by strengthening international relationships, supporting new research collaborations, and attracting high-quality research from across the globe. IJMHS is putting in place a number of initiatives to enhance the attractiveness, visibility, and impact of the Journal. This includes: the planning of thematic series; introducing a broader range of article categories; utilising the Journal as the primary method of dissemination of work completed under the auspices of the International Observatory of Mental Health Systems [33,34]; and, particularly, encouraging submissions from authors from countries and regions not yet well represented in the journal. IJMHS is well positioned to build on the first six years of publication in the context of increasing global attention to and support for global mental health research [35].

## Competing interests

Three of the authors (HM, AW and RK) are editors of the journal.

## Authors’ contributions

HM conceived the project and wrote the first draft. All authors contributed to data analysis and interpretations and to the writing of subsequent drafts. All authors have seen and approved the final version of the manuscript. All authors read and approved the final manuscript.
